# Supplementary material for: Factors associated with coronary heart disease in COPD patients and controls
Source: PLoS One. 2022 Apr 27;17(4):e0265682. doi: 10.1371/journal.pone.0265682 (PMC9045629; doi:10.1371/journal.pone.0265682)
Supplement: Attachment — Submitted filename: Response letter draft.docx [file pone.0265682.s001.docx]

Response letter

**Reviewer 1**

Reviewer #1: Svedsen and colleagues analyse the presence of coronary artery plaques and calcium score, respectively, in patients with COPD compared with controls without CPOD in a Norwegian cohort, with focus of analysis on potential risk factors for these outcomes among the patients with COPD. As outlined nicely in the paper, this topic is of interest given the large co-occurrence of cardiovascular disease and COPD and the relevance for morbidity and prognosis. Reading the paper, I had some comments and suggestions for strengthening the paper:

We thank the reviewer for these comments and hope we have addressed all comments and suggestions adequately.

- A general comment is that the analysis in fact focuses on a range of potential predictors of the outcomes (stenosis and calcium score) - not only COPD phenotype. Suggest that is reflected in the title ('Factors associated with' or 'Predictors of ...' instead of COPD phenotype), especially as many of the findings not pertain closely to phenotype, rather factors more broadly.

We suggest changing the title to:

"Factors associated with coronary heart disease in COPD patients and controls"

It also takes a while in Methods before the phenotype variables are introduced.

We find that it is natural to first describe the study design, before data collection; in which paragraph we find it natural to present collection of all variables, not only COPD disease characteristics (or phenotypes). Thus, we are a little uncertain what the reviewer has in mind with this comment.
Please revise (and clarify) the aim accordingly (now the aim is rather vague and differ in Abstract and the main text).

We agree, re-reading our statement on aims, they could be better formulated. In the abstract the aim of seeing whether COPD patients had different risk than controls was left out due to word restraints, which may be a poor excuse.

We suggest changing the aims at the end of the introduction to: "The aim of this study was to see whether COPD patients did have a true higher risk for CHD than subjects without COPD, and to examine a range of potential factors associated with CHD in COPD patients and controls", and if the editor allows, we suggest keeping the exact same wording in the abstract.

- Need to be clearer on the limitation in terms of sample size / power. Many well established risk factors for cardiovascular disease (CVD) did not "fall out" in the analysis -- however, this is not to be interpreted as these factors are not risk factors in COPD patients (COPD patients are "spared") - when, in fact, it is the other way around (not implying this is stated by the authors, it is not), but important that this limitation is made clear. Insufficient power likely to explain the absence of a lot of expected associations? - that also is a prolem for the relevance of the analysis as a whole, but the analysis is still valuable as it is well done and could be combined with other datasets for meta-analyses
We agree, the chance of a type II error due to lack of study power is a possibility, and this could have been stated more clearly.

Re-reading our manuscript, we think our second point (Second, the outcomes coronary artery stenosis and CaSc > 100 are common events in the population, and it is important to realize that odds ratios do not approximate relative risk in this circumstance, which means that the effect size must be interpreted with caution.) in the limitations discussion was perhaps of less value to the reader (too general a statement), and suggest replacing the second point with the more important limitation on power/sample size:

"Second, study sample size may have been too low, and there is a chance for type II errors. Thus, we urge caution in interpreting all negative associations as proof of a non-causal relationship."

- Factors in the models: please state how adjustment factors as well as factors to evaluate in the model were selected.
The second reviewer had the same question ("In the second part, logistic regression uses different COPD characteristics as independent variables.
How were the explanation variables chosen? A priori based on subject knowledge matter or based on unadjusted analyses?")

As can be seen from tables 2 -> 5, pretty much all variables from the unadjusted analyses were included in the multivariable analyses, thus we chose variables based on our a priori beliefs of what may be interesting/potentially having an effect, not p-values from the unadjusted analyses. However, in some instances two categories may have effects/explanatory power with an unacceptable or uninterpretable degree of co-linearity, and only one variable of the two were included in the multivariable analyses.

For instance, tobacco smoking can be difficult to determine how best to adjust for. We have the categorical variable never, ex, current; where only controls have actual never smokers, thus a cell with zero for COPD patients, and we have pack years smoked, which is zero of course for the exact same control individuals who are never smokers. As can be seen from table 3, neither variables significantly predicted the presence of coronary stenosis (in our humble opinion likely due to power - an issue both reviewers have pointed out). Yet, we think we cannot exclude such an important variable from the multivariable analyses, and choose pack years, as we believe we then retained most of the potential explanatory power from that variable. (And of course, we have run all models exchanging pack years with smoking as a categorical variable and find pretty much the same results). Use of statins and measured cholesterol is another example, where use of statins so heavily influence measured cholesterol, that we think use of statins was the better variable to include in the multivariable models.

- Intro: states that COPD severity is graded based on lung function -- not sure that statement is entirely correct any more, please revise

We suggest changing the wording to: "COPD disease severity is related to lung function decline, yet we know degree of emphysema can vary considerably between to patients with the same lung function."

- Aim: as mentioned above, make the aims similar and specific - the aim in the paper is not good - "... whether different COPD patients had an increased risk..." needs revision in terms of scientific contents and language (surely different patients will have different risks, it would be surprising if all COPD patients had the same risk, etc) - evaluate factors associated with... ?
As stated above, we have re-written the aims and hope they are now adequate: " The aim of this study was to see whether COPD patients did have a true higher risk for CHD than subjects without COPD, and to examine a range of potential factors associated with CHD in COPD patients and controls."

- Methods: please state which patients that were included in the used databases - now it only says that 16 asthma patients were included (which was confusing at the first read); inclusion and exclusion criteria. Suggest to move the data on actually included number of participants to the start of Results.
To understand the study design, we find it is most always wise to be specific in the methods, not wait to the results. However, we agree the description was difficult to follow; this was pointed out also by Reviewer 2. Per reviewer 2's suggestion we have included a flow chart; and also we have changed the wording ono the study design, which we hope makes it easier for the reader.

- Why was the CaSc threshold set to 100 for the outcome?

No certain consensus exists to our knowledge on which cut-off to use, some use 10, some 100 and some 400. Either choice is based on Agatson, and the higher the score the higher the risk. It is our feeling from the current literature, that 100 is most often used; and we think it makes sense based on tis nice review paper from 2010: <https://www.ncbi.nlm.nih.gov/pmc/articles/PMC2922319/> - note Table 3 in the paper. As defined by Rumberger et al in 1999 (https://pubmed.ncbi.nlm.nih.gov/10089993/), CaSc > 100 correlates with a high risk for coronary heart disease, and this is the level of risk we think it makes sense to try to predict in our study sample. We realize from the reviewer's question we had not defined this clearly in our original draft. Thus, we suggest adding the following sentence in the methods under the CT protocol section: "As defined by Rumberger et al (21), a CaSc > 100 was used as an indicator of a high risk for coronary heart disease." See also our reply below regarding correlation between CaSc and stenosis.

- Tables: no need to include both the "yes" and the "no" categories for binary variables

The "No" category has been removed in the revised Table 2.

- Methods and Results: how were the models specified to begin with (in terms of factors evaluated and adjusted for) and how were the final factors to report determined?
See answer to the same point above.

- How many had a SaSc >500 (and were excluded from CTA)? Potential influence on findings?
139, of which 85 were COPD patients. Most likely, many of those would have coronary stenosis had they been examined. Thus, excluding them – as the models with coronary stenosis assessment do by default – and since a little more COPD patients than controls were excluded, this restriction will perhaps underestimate the effect of having COPD. This is precisely why we choose to also model risk of having a higher CaSc, as we think that supplements the direct assessment of visible coronary stenosis.

We suggest adding the information about 139 & 85 in the methods so that the sentence regarding calcium score > 500 in the revised manuscript now reads: ”The CCTA was not performed if the CaSc was higher than 500 (n=139, of which 85 were COPD patients)”

- Table 4: "for the chance of finding..." - please revise and specify
We suggest changing the Table heading to : “The odds ratios (95% CI) for having significant coronary stenosis on CT angiography (n=574)”

- One wonders how good CaSc is as outcome - how was the correlation within the outcomes (CaSc with stenosis)? Would be good with more data on that, including in Methods as rationale for the cut-off and use of that outcome

Among 561 subjects with non-significant stenosis, the median (IQR) CaSc was 15 (0-105), among 46 subjects with significant stenosis it was 243 (61-391). P-value = 0.0001, Kruskal Wallis test. Thus, the correlation is very strong although imperfect, but CaSc captures many more subjects with likely subclinical stenosis and/or at risk.
We suggest adding this information to the methods section: "Among 561 subjects with non-significant stenosis, the median (IQR) CaSc was 15 (0-105), whereas among 46 subjects with significant stenosis the median CaSc was 243 (61-391), p-value = 0.0001, Kruskal Wallis test. As defined by Rumberger et al (21), a CaSc > 100 was used as an indicator of a high risk for coronary heart disease."

- Please revise the sentence "Since this is a cross-sectional study, we can only speculate", as well as the sentences with "which can easily be interpred as", and "makes it uncertain to establish"

We suggest removing the sentence including the word "speculate", as the sentence itself does not really add information, as well as the part of the other sentence.

For the second sentence regarding causality and cross-sectional studies, we think it is worth point it out, although it may be obvious to many readers. We suggest re-phrasing to: "First, Causality cannot be established from a cross-sectional study".

**Reviewer 2**

Reviewer #2: The main purpose of this study was to investigate it if different COPD characteristics or phenotypes are associated with an increased risk of coronary heart disease. The conceptualization is really interesting and the overall planning of the study is sound and clear. I have no major concerns but a number of minor comments mainly about design and analyses.
We thank the reviewer for these kind comments, and hope we have been able to address the interesting points raised below:
1. Design and data collection
The design and data collection could be more clearly described. The title says case control study, but is it really? The study has two parts, the first investigating the prevalence of coronary atherosclerosis in patients with COPD and non-COPD controls and the second investigating the associations of specific COPD characteristics with the dependent variable of CHD. However, it is not like the groups are defined from the outcome CHD and compared about the risk factors and it is unclear how the controls were chosen. The authors refer to the original papers of the involved cohorts, but it is unclear from the present paper if matching of cases and controls have been performed. As for the second part, it is unclear if the independent variables are collected at the same time as the outcome variable or not. The paper would benefit a lot from a flow chart clarifying the design, timing, the contributions from different other cohorts, and the attritions.
We understand that the description of the design was difficult to understand, as we are using patients from two cross-sectional samples, one of which (the GeneCOPD follow-up) was the second phase of a follow-up study.

We have tried to improve this part, by adding text to the methods description of the study design and adding a new Figure 1 with a flow chart per the reviewers' suggestion.

2. Comparison of CHD in COPD and non-COPD
The first major conclusion is that COPD patients are more likely to have objective measures of CHD. This is not surprising as the COPD patients are older, have more pack years and more diabetes. Have you considered comparing the groups by propensity score matching?

No, truth be told, we have little experience using propensity score matching. It is our understanding of PSM that it is an experimental technique whereby use of an artificial control group allows theoretically modelling the effect of a hypothetical intervention. We can see how this may be an alternative to traditional regression modelling, but we do not really understand how this would better fit our data since PSM also is based on a logistic model. But we may miss something here, as PSM is outside our modelling comfort zone. We certainly agree that age, smoking, and diabetes are risk factors for coronary heart disease, in both non-COPD subjects and patients with COPD. Our traditional modelling implies to us that COPD has added risk, after adjustment for the aforementioned variables. Since we have used a traditional modelling that most researchers use, and are familiar with (logistic regression), we believe that makes interpretation and acceptance better for a wider audience. However, we are the first to admit our lack of PSM modelling experience, and thus, if the reviewer can point us toward similar published studies using PSM or explain how the reviewer envisions using PSM on our data, we will of course try to look into it.

3. Logistic regression
In the second part, logistic regression uses different COPD characteristics as independent variables.
How were the explanation variables chosen? A priori based on subject knowledge matter or based on unadjusted analyses?

As can be seen from tables 2 -> 5, pretty much all variables from the unadjusted analyses were included in the multivariable analyses, thus we chose variables based on our a priori beliefs of what may be interesting/potentially having an effect, not p-values from the unadjusted analyses. However, in some instances two categories may have effects/explanatory power with an unacceptable or uninterpretable degree of co-linearity, and only one variable of the two were included in the multivariable analyses.

For instance, tobacco smoking can be difficult to determine how best to adjust for. We have the categorical variable never, ex, current; where only controls have actual never smokers, thus a cell with zero for COPD patients, and we have pack years smoked, which is zero of course for the exact same control individuals who are never smokers. As can be seen from table 3, neither variables significantly predicted the presence of coronary stenosis (in our humble opinion likely due to power - an issue both reviewers have pointed out). Yet, we think we cannot exclude such an important variable from the multivariable analyses, and choose pack years, as we believe we then retained most of the potential explanatory power from that variable. (And of course, we have run all models exchanging pack years with smoking as a categorical variable and find pretty much the same results). Use of statins and measured cholesterol is another example, where use of statins so heavily influence measured cholesterol, that we think use of statins was the better variable to include in the multivariable models.

I find the research question very relevant, but it would have been even more interesting with more COPD characteristics as markers for systemic inflammation or other phenoptypes. Was chronic bronchitis or eosinophilic COPD investigated as independent variables?

No, but we realized reading these comments, that we should have for eosinophilic inflammation, where treatment effects on COPD from inhaled steroids may differ between eosinophlilic and non-eosinophilic COPD. Chronic bronchitis is usually defined as having chronic cough daily for 3 months or more during a year, and unfortunately, we do not have standardized information on symptomatology for chronic cough with phlegm collected for all study subjects.

However, we have eosinophil cell counts taken on the same day as CRP and lung function testing, and we have updated all tables with this included information. (Currently, there are two different common definitions in the literature, absolute eosinophil cell count above a threshold - often 0.3*10ˆ9 cells/L - or as a percentage. In our data with 0.3 or higher as cut off, we see 24% and 36%, whereas using ≥2% we have 64 and 67% We believe the absolute count better fits the data, and makes biological sense (A certain level is likely biologically active), and thus we have used the 0.3 threshold.

Emphysema was defined as 10% of the lungs or not, what happens if the analyses are repeated using emphysema a continuous score as independent variable?

Univariately, emphysema score (% of lung with < 950 Hu) was nonsignificant as a predictor of coronary stenosis in COPD patients (mean 2.7% among those with stenosis versus those without), but significantly associated with Calcium Score (mean 3.5% among those with CaSc > 100, versus 0.9% among those with CaSc < 100, p<0.001). In the regression analyses, emphysema was nonsignificant in both models, and all other associations pretty much unchanged (in the CaSc prediction model, use of ACE/ARBs had p-value changing from 0.055 to 0.047). Use of emphysema as a continuous variable probably has more value in terms of being adjusted for, much the same as pack-years smoked, and thus we suggest exchanging the emphysema variable from no/yes to a continuous score per the reviewers' suggestion.

And was both hypoxemia and hypercapnia analysed?

Among the COPD patients who had pO2 < 8 kPa, 16 also had pCO2 > 6 kPa. Modeling with for instance a 3-category variable 1=no respiratory failure, 2= respiratory failure type I, 3=respiratory failure type II, we find no added signal or explanatory value to the original modelling of respiratory failure yes/no based in hypoxemia. It is difficult to disentangle the potential effects of pCO2 and pO2, and likely having pCO2 higher may more than anything be a sign of increased severity of COPD, also measured by some of the other variables like GOLD and emphysema score, thus we suggest keeping the original modelling using only pO2.

ACO? Non-smoking COPD? COPD with early onset or exposure during childhood/premature birth?

These are variables we do not really have good access to - as can be seen by Table 1, there was almost non COPD patients whom were never smokers in our cohorts, and for ACO we had no standadized definition to define COPD patients as ACO or not, rather we spent some time making sure they did not have asthma in our opinion, and exclude them from the COPD group if they we thought they had asthma (very few did). And finally, we do not have data on childhood exposures or birth weight. As interesting as that may have been, the age range of our COPD patients were from 49 to 90 years.

It would have been of great interest if the analyses did not only adjust for different characteristics/phenotypes, but also included stratification and interaction analyses to see if there is an effect modification by phenotype, ie- if factors associated with CHD differs between different phenotypes.

The multivariable models are large, adding many variables, which "strain" the models to some degree, however, being robust in that adding or removing variables make little or no change to the signal we see, with no change in which variables come out statistically significant and which do not.

However, running all first-order interactions against each other in both models presented in Table 5a & b, would mean testing 144 associations. We find it difficult to justify testing all 144 associations, for instance it is hard to argue (to us) for an a priori likelihood that the hypothetical effect of eosinophilic COPD differed in patients with or without diabetes.

Choosing instead to look at sex, age and smoking since these are (arguably) the most important biologically explanatory variables for coronary heart disease, we tested all first order-interactions between these three variables and all other, a total of 66 associations. With this number of tests, we set a p-value to 0.01, and zero statistically significant interactions were found. We suggest adding this information as a sentence in the methods section: "First-order interactions between sex, age, smoking and all other variables were tested for the COPD patients. Due to the large number of interactions (n=66), a *p*-value of 0.01 was used, for all other analyses a *p*-value of < 0.05 was considered statistically significant.", and the following addition in the results section:" .. , and neither of the first-order interactions tested were statistically significant (data not shown). "

4. Power analysis
The second major conclusion is that no specific COPD characteristics were independently associated with a higher risk for CHD. Could this be a type 2 error? Was there a power analysis performed to ensure a reasonable number of participants?

No, as pointed out also by reviewer 1 ("Need to be clearer on the limitation in terms of sample size / power."), there is of course a limitation in that lack of power could be a reason non-significant results were found. As stated in our response to reviewer 1, we have added to the discussion more on this limitation. As for power calculations, none were performed on this specific research question prior to this study. Many variables were found to be potentially interesting as potentially associated/explanatory here, and performing power calculations on a hypothetical multivariable model is quite difficult and uncertain.

We suggest adding to the limitations section the following paragraph: "Second, study sample size may have been too low, and there is a chance for type II errors. Thus, we urge caution in interpreting all negative associations as proof of a non-causal relationship."

5. Attrition
Was there any attrition analysis?

This being a non-randomly drawn cross-sectional sample, were we used what we had, not really, no.

From a potential pool of 910 COPD and/or controls, 775 had some sort of coronary disease variable assessment (stenosis or CaSc) and 607 had both stenosis and CaSc assessed. Thus, we can compare how these three samples look in terms of age, sex, smoking and body composition for instance (see Table below). Age is somewhat lower in the last sample (with both stenosis and CaSc assessment), perhaps not surprisingly. However, to what degree - if any - this relates to more or less external validity we feel unsure. And, as the manuscript already is bursting with results, we suggest not adding this analyses to the manuscript, although we will of course reconsider if the reviewer finds it important.

| **Three different samples** | |  |  |  |  |  |
| --- | --- | --- | --- | --- | --- | --- |
|  | n=910 (all - 16 asthma) | | n=775 (all with some measure of coronary disease) | | n=607 (all having both coronary stenosis assessment and CaSc) | |
|  | n | % | n | % | n | % |
| *Sex* |  |  |  |  |  |  |
| Women | 407 | 44.7 | 353 | 45.6 | 303 | 49.9 |
| Men | 503 | 55.3 | 422 | 54.5 | 304 | 50.1 |
| *Age categories (years)* |  |  |  |  |  |  |
| 40 - 59.9 | 206 | 22.7 | 190 | 24.5 | 183 | 30.2 |
| 60 - 69.9 | 394 | 43.3 | 334 | 43.1 | 278 | 45.8 |
| 70 - 90 | 309 | 34.0 | 251 | 32.4 | 146 | 24.1 |
| *Body composition* |  |  |  |  |  |  |
| Normal | 621 | 68.2 | 529 | 68.3 | 437 | 72.0 |
| Cachectic | 142 | 15.6 | 121 | 15.6 | 80 | 13.2 |
| Obese | 147 | 16.2 | 125 | 16.1 | 90 | 14.8 |
| *Smoking habits* |  |  |  |  |  |  |
| Never | 40 | 4.4 | 20 | 2.6 | 16 | 2.6 |
| Ex | 589 | 64.7 | 505 | 65.2 | 385 | 63.4 |
| Current | 281 | 30.9 | 250 | 32.3 | 206 | 33.9 |
